# Primary diffuse cutaneous plasmacytoma: when a correct clinico-pathologic approach is mandatory for the patient's health^☆^^☆☆^

**DOI:** 10.1016/j.abd.2019.04.004

**Published:** 2019-11-07

**Authors:** Giuseppe Broggi, Enrica Martino, Valeria Calafiore, Rosario Caltabiano

**Affiliations:** a Department Gian Filippo Ingrassia, Section of Anatomic Pathology, University of Catania, Catania, Italy; b Division of Hematology, Azienda Ospedaliero-Universitaria Policlinico-Vittorio Emanuele, Catania, Italy

Dear Editor,

A 76-year-old woman presented with multiple purplish plaques located on the arms (Fig. 1), deltoid region, elbows, wrist, mammary region, and legs. Lesions were painful at the touch without itching. Clinically, a diagnosis of eczema was suspected and a skin biopsy of the left arm was conducted.

**Figure 1:**
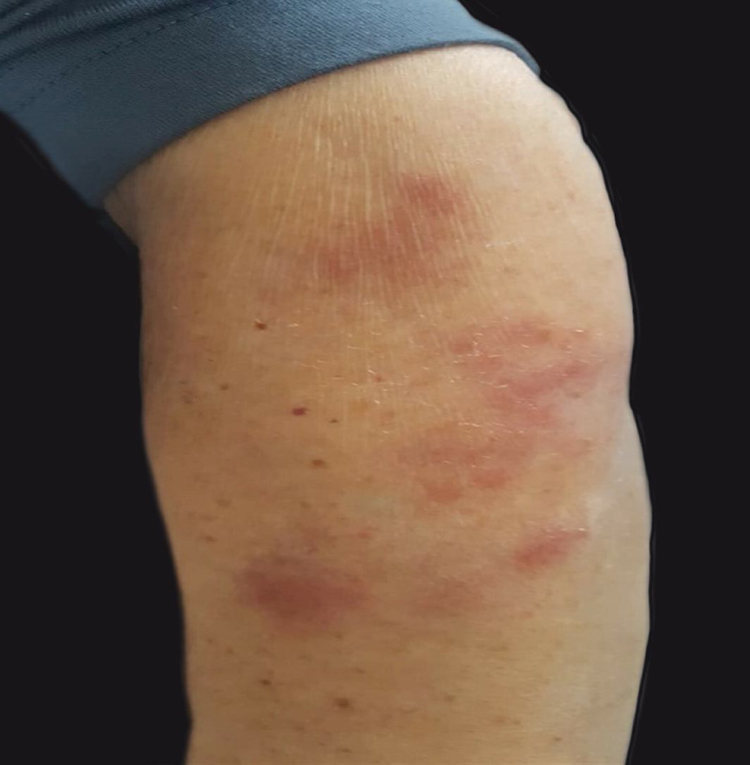
Multiple, painful, non-itching, purplish cutaneous plaques located on the left arm.

Histological examination of hematoxylin & eosin-stained sections showed the presence of diffuse clusters of atypical oval-shaped cells with abundant cytoplasm, eccentric nuclei, “clock face” chromatin, and sometimes prominent nucleoli, infiltrating the medium and deep dermis (Fig. 2). Mitotic figures were seen. Neoplastic cells were morphologically similar to mature plasma cells, so a specific immunohistochemical panel was performed: they were diffusely positive for CD79a, CD138, CD56, MUM-1, and EMA, and totally negative for CD20. Immunohistochemical studies for kappa and lambda light chains revealed a monoclonal expression of immunoglobulin kappa lights chains (Fig. 3).

**Figure 2:**
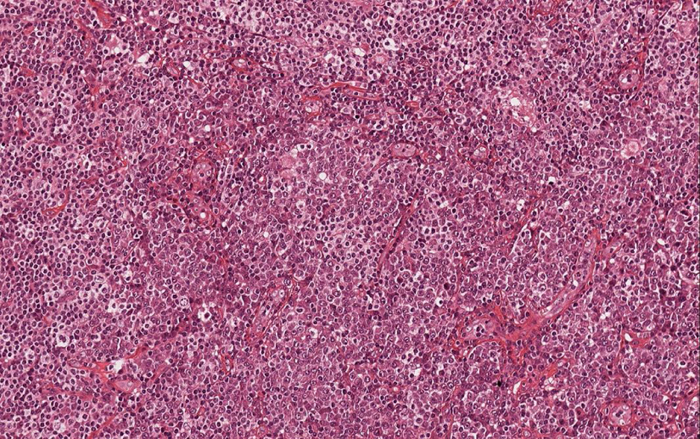
Clusters of oval-shaped cells with abundant cytoplasm, eccentric nuclei, and “clock face” chromatin (Hematoxylin & eosin, x200).

**Figure 3:**
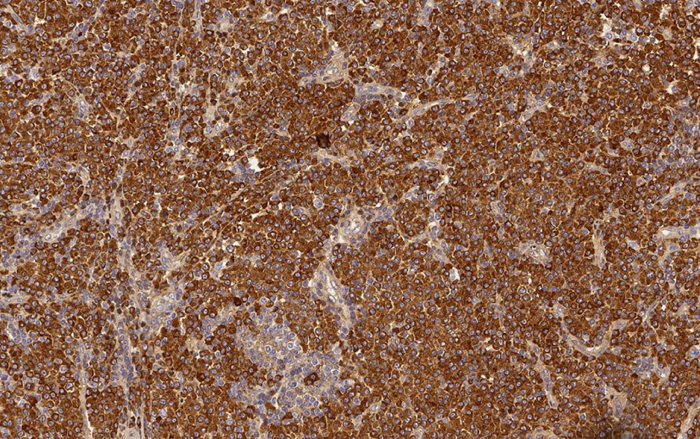
Monoclonal expression of immunoglobulin kappa light chains (Immunoperoxidase, x200).

To complete the diagnostic process, a bone marrow biopsy was performed; it was negative for multiple myeloma (MM) localization (less than 10% plasma cells; no clonal restriction). There were no Bence-Jones proteins in the urine. Hemogram and biochemical blood analysis revealed a normal value of hemoglobin and normal serum creatinine and calcium. Serum protein electrophoresis highlighted a lambda light chain spike.

Once the absence of other sites of disease was confirmed, a clinico-pathologic diagnosis of primary diffuse cutaneous plasmacytoma (PDCP) was rendered. Considering the extensive dissemination of the cutaneous involvement, the patient received systemic therapy. It consisted of bortezomib at the dosage of 1.3 mg/m^2^ subcutaneously at day 1, 8, 15, and 22, melphalan given orally at the dosage of 14 mg at day 1, 2, 3, and 4, and dexamethasone at the dosage of 20 mg at day 1–2–8–9–15–16–22–23 (regimen). After nine cycles, fluorodeoxyglucose positron emission tomography–computed tomography showed complete disappearance of the skin lesions and absence of the lambda immunoglobulin G spike at the serum protein electrophoresis.

The patient completed therapy without adverse effects and, to date, after one year and eight months of follow-up, no recurrence of disease has been detected.

PDCP is a rare disease^1^ that arises primarily in the skin, so it can be considered as a localized cutaneous extramedullary plasmacytoma (EMP) and should not be confused with secondary cutaneous plasmacytoma (SCP) in the context of MM.^2^ According to a recent systematic review, only 68 cases of primary cutaneous plasmacytomas (PCPs) have been reported in literature, the majority of which were solitary lesions.^3^

PDCP usually arises as purplish-blue cutaneous nodules with a predilection for the face, trunk, and extremities.^1^, ^2^ Histologically, a diffuse or nodular infiltration pattern can be recognized in PCPs and neoplastic cells may show different stages of plasma cells maturation process, from well differentiated to pleomorphic (similar to plasmablasts) features.^4^, ^5^ PCPs with plasmablast-like features are composed of neoplastic cells with higher nuclear/cytoplasmic ratio, finely dispersed chromatin, and more prominent nucleoli.^4^ Epidermotropism is usually absent in PCPs.^5^

The main prognostic factor is clinical presentation (solitary *vs.* multiple lesions),^3^ but is also important to consider patient's performance status and comorbidities which can impair compliance in the treatment. Tsang et al., in their systematic review,^3^ showed that the only clinical variable associated with recurrence free survival (RFS) and overall survival was the number of lesions (solitary *vs*. multiple); in particular, they observed a large difference in median survival and RFS between patients with solitary lesions and those with multiple lesions.^3^ In the latter subset of patients, systemic chemotherapy is necessary due to the high rate of evolution to MM and the poor prognosis.^1^

Differential diagnosis of PCP includes MM with SCP, EMP with secondary cutaneous involvement, other B-cell lymphomas of the skin, in particular marginal-zone lymphoma with marked plasma cell differentiation, and infective diseases such as *Borrelia* infections.

It is crucial to emphasize that neoplastic plasma cells in PCPs can be cytologically indistinguishable from reactive ones in infectious diseases, representing a potential diagnostic pitfall for pathologists, thus immunohistochemical evaluation of mono- or polyclonal expression of immunoglobulin light chains,^4^ combined with the absence of an evocative history of infection or causal agent identification, are crucial for a diagnosis of malignancy.^5^

MM rarely involves the skin and because of the absence of distinctive histological features, only with clinical and laboratory examinations is it possible distinguish between SCP in MM and PCP.^2^

Finally, PCDP is a rare disease that requires a wide multidisciplinary approach, which is strongly recommended to achieve a certain diagnosis of “true” PCP, in order to choose the optimal treatment.

## Financial support

None declared.

## Authors’ contribution

Giuseppe Broggi: Approval of the final version of the manuscript; conception and planning of the study; composition of the manuscript.

Enrica Martino: Conception and planning of the study; composition of the manuscript.

Valeria Calafiore: Collection, analysis, and interpretation of data; intellectual participation in the propaedeutic and/or therapeutic conduct of the studied cases.

Rosario Caltabiano: Approval of the final version of the manuscript; critical review of the manuscript.

## Conflicts of interest

None declared.
